# Prognostic Relevance of Cardiopulmonary Exercise Testing for Patients with Chronic Thromboembolic Pulmonary Hypertension

**DOI:** 10.3390/jcdd9100333

**Published:** 2022-10-01

**Authors:** Ralf Ewert, Till Ittermann, Delia Schmitt, Elena Pfeuffer-Jovic, Johannes Stucke, Kristin Tausche, Michael Halank, Jörg Winkler, Andreas Hoheisel, Beate Stubbe, Alexander Heine, Hans-Jürgen Seyfarth, Christian Opitz, Dirk Habedank, Roland Wensel, Matthias Held

**Affiliations:** 1 Internal Medicine B, Pneumology, University Hospital Greifswald, 17475 Greifswald, Germany; 2 Department of Community Medicine, University Hospital Greifswald, 17489 Greifswald, Germany; 3 Department of Internal Medicine, Medical Missio Hospital, 97074 Würzburg, Germany; 4 Internal Medicine, Pneumology, University Hospital Dresden, 01307 Dresden, Germany; 5 Medical Practice, 04103 Leipzig, Germany; 6 Department of Pneumology, Medical Center, Faculty of Medicine, University of Freiburg, 79106 Freiburg, Germany; 7 Internal Medicine, Pneumology, University Hospital Leipzig, 04103 Leipzig, Germany; 8 Internal Medicine, Cardiology, DRK-Hospital Berlin, 14050 Berlin, Germany

**Keywords:** all-cause mortality, balloon pulmonary angioplasty, cardiopulmonary exercise testing, chronic thromboembolic pulmonary disease, chronic thromboembolic pulmonary hypertension, comorbidities, lung function, prognosis, pulmonary endarterectomy, therapy

## Abstract

Background: Following acute pulmonary embolism (PE), a relevant number of patients experience decreased exercise capacity which can be associated with disturbed pulmonary perfusion. Cardiopulmonary exercise testing (CPET) shows several patterns typical for disturbed pulmonary perfusion. Research question: We aimed to examine whether CPET can also provide prognostic information in chronic thromboembolic pulmonary hypertension (CTEPH). Study Design and Methods: We performed a multicenter retrospective chart review in Germany between 2002 and 2020. Patients with CTEPH were included if they had ≥6 months of follow-up and complete CPET and hemodynamic data. Symptom-limited CPET was performed using a cycle ergometer (ramp or Jones protocol). The association of anthropometric data, comorbidities, symptoms, lung function, and echocardiographic, hemodynamic, and CPET parameters with survival was examined. Mortality prediction models were calculated by Cox regression with backward selection. Results: 345 patients (1532 person-years) were included; 138 underwent surgical treatment (pulmonary endarterectomy or balloon pulmonary angioplasty) and 207 received only non-surgical treatment. During follow-up (median 3.5 years), 78 patients died. The death rate per 1000 person-years was 24.9 and 74.2 in the surgical and non-surgical groups, respectively (*p* < 0.001). In age- and sex-adjusted Cox regression analyses, CPET parameters including peak oxygen uptake (VO_2_peak, reflecting cardiopulmonary exercise capacity) were prognostic in the non-surgical group but not in the surgical group. In mortality prediction models, age, sex, VO_2_peak (% predicted), and carbon monoxide transfer coefficient (% predicted) showed significant prognostic relevance in both the overall cohort and the non-surgical group. In the non-surgical group, Kaplan–Meier analysis showed that patients with VO_2_peak below 53.4% predicted (threshold identified by receiver operating characteristic analysis) had increased mortality (*p* = 0.007). Interpretation: The additional measurement of cardiopulmonary exercise capacity by CPET allows a more precise prognostic evaluation in patients with CTEPH. CPET might therefore be helpful for risk-adapted treatment of CTEPH.

## 1. Background

Patients who survive acute pulmonary embolism (PE) commonly have impaired physical capacity and reduced quality of life [1]. The term “post-PE syndrome” has been coined to describe these findings [2,3]. Diagnosis requires comprehensive evaluation [2]. A highly variable proportion of these patients (3–15%) shows disturbed pulmonary perfusion with or without pulmonary hypertension (PH), known as chronic thromboembolic PH (CTEPH) [4,5,6] and chronic thromboembolic pulmonary disease (CTEPD), respectively. Patients with CTEPD have normal pulmonary artery pressure at rest, but may develop hemodynamic abnormalities under exercise, similar to those observed in CTEPH [7,8].

Cardiopulmonary exercise testing (CPET) is a valuable tool to measure functional impairment in patients with persisting symptoms following PE. CPET has a high sensitivity to detect pulmonary perfusion defects [9,10,11] and may therefore help to identify patients for further invasive diagnostic procedures. Patients with CTEPD show a pattern of ineffective ventilation and gas exchange disturbance with reduced peak oxygen uptake (VO_2_peak), elevated alveolar–arterial oxygen gradient (P[A-a]O_2_), and elevated arterial to end-tidal carbon dioxide gradient (P[a-ET]CO_2_) under exercise compared with control patients with dyspnea in the absence of PH or pulmonary vascular disease [7]. Different CPET patterns are also helpful to discriminate patients with CTEPH from symptomatic patients without pulmonary vascular disease [9,12] and patients with other forms of PH [13,14]. In addition, CPET can be used to assess the severity of CTEPH [15,16]. CPET is explicitly recommended as a comprehensive tool for the follow-up of patients with PE by the current European Respiratory Society (ERS) statement on the diagnosis and treatment of CTEPH [17].

The analysis of mixed cohorts of patients with CTEPH and pulmonary arterial hypertension (PAH) suggests that CPET can also provide relevant prognostic information in patients with CTEPH [18,19]. Thus, CPET might be a comprehensive tool to guide treatment decisions. We therefore performed a multicenter outpatient study to assess the prognostic value of CPET-derived parameters in patients with CTEPH.

## 2. Study Design and Methods

The study has been approved by the ethics committee of the University of Greifswald (Registration Number BB 216/20, 17 November 2020). All patients gave written informed consent.

### 2.1. Patients

**Figure 1:**
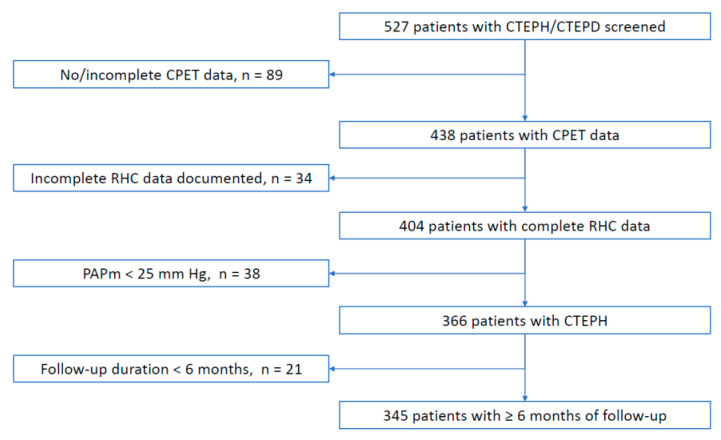
Study flow-chart.—Patient flow. CPET = cardiopulmonary exercise testing; CTEPD = chronic thromboembolic pulmonary disease; CTEPH = chronic thromboembolic pulmonary hypertension; PAPm = mean pulmonary artery pressure; RHC = right heart catheterization.

We retrospectively reviewed medical records of 527 patients with CTEPH/CTEPD from seven PH specialist outpatient departments across Germany between 2002 and 2020. The main inclusion criterion for this analysis was CTEPH confirmed by tertiary PH expert centers according to the current guidelines [20]. We excluded patients with incomplete hemodynamic and/or CPET data (n = 123), mean pulmonary artery pressure (PAPm) < 25 mmHg (n = 38), or an observation period of less than 6 months, allowing 345 patients to be analyzed (Figure 1).

All patients were evaluated by an experienced high-volume CTEPH surgeon for pulmonary endarterectomy (PEA) or balloon pulmonary angioplasty (BPA). Patients who received one of these procedures are hereafter referred to as the surgical group. All patients who were not suitable for PEA or BPA (non-surgical group) were treated with optimal medical therapy.

Patients’ gender, age, and body mass index (BMI) were documented, and comorbidities were analyzed according to the patients’ charts. Functional and clinical characterization was based on World Health Organization (WHO) functional class, 6-min walk distance (6-MWD), and lung functional, echocardiographic, hemodynamic, and CPET data.

### 2.2. Lung Function and CPET

All technical staff in the participating centers underwent specific lung function and CPET training.

Lung function parameters were calculated according to normal values as described previously [21,22,23]. Obstructive pulmonary disease was defined as forced expiratory volume in 1 s (FEV1)/forced vital capacity (FVC) < 70%, restrictive pulmonary disease as total lung capacity (TLC) < 80%, and clinically relevant diffusion impairment as diffusion capacity of the lung for carbon monoxide (DLCO) < 60% of normal.

Symptom-limited CPET was performed using a calibrated electromagnetically braked cycle ergometer (Ergoselect 100; Ergoline, Bitz, Germany, or E-bike basic Plus; GE Medical Systems, Solingen, Germany), with a resting period of 2–3 min followed by unloaded pedaling over 1–2 min and a subsequent exercise protocol consisting of either a ramp load (5–10–12.5 W increase/min) or incremental increases (16 W increase/min) until exhaustion. Gas exchange was measured using an Oxycon Pro or Master Screen CPX with a Rudolf mask (Jäger/Viasys Healthcare, Hoechberg, Germany). Gas exchange parameters including oxygen uptake (VO_2_), end-tidal partial pressure of carbon dioxide (PETCO_2_), end-tidal partial pressure of oxygen (PETO_2_), and carbon dioxide production (VCO_2_), as well as minute ventilation (VE), tidal volume, and respiratory rate, were assessed using breath-by-breath analysis.

Graphical presentations were generated as Wassermann 9-panel plots using computer-averaged 10 s intervals. VO_2_peak was defined as the highest VO_2_ before the end of the exercise. The maximum voluntary ventilation (MVV) was calculated using a factor of 40 (MVV40 = FEV1 × 40) [24]. To calculate P(A-a)O_2_ and P(a-ET)CO_2_, capillary blood gases were obtained from hyperemic earlobes at rest and at peak exercise. The anaerobic threshold (ventilatory threshold 1, VT1) was determined according to the recommendations of the German working group on CPET [25]. The calculation of CPET data was performed centrally using standard reference values [26].

### 2.3. Right Heart Catheterization and Echocardiography

Right heart catheterization (RHC) was performed according to the guidelines of the European Society of Cardiology (ESC)/ERS [20] and German recommendations [27].

Resting echocardiography was performed by experienced physicians according to relevant guidelines [28,29]. Tricuspid regurgitation was classified according to American College of Cardiology/ESC [28,30] recommendations, and right ventricular systolic pressure (RVSP) was estimated by simplified Bernoulli equation via tricuspid regurgitation velocity (v) as RVSP (mm Hg) = 4v^2^, with the addition of 5 mm Hg if the inferior vena cava was not dilated and there was visible respiratory variability, and 10 mm Hg if the inferior vena cava was dilated or without respiratory variability.

### 2.4. Follow-Up

All patients were seen during routine follow-up or contacted by telephone. The date of evaluation was 31 December 2020.

### 2.5. Statistics

Continuous variables were reported as mean ± standard deviation (SD) and categorical variables as absolute frequencies and percentages. Differences between the surgical (PEA/BPA) and non-surgical (medical therapy only) groups were examined by the Wilcoxon test for continuous data and by Fisher’s exact test for categorical data. Survival analyses included Kaplan–Meier curves as well as Cox regression models adjusted for age and sex. Results are expressed as hazard ratios (HR) and 95% confidence intervals (CI). Mortality prediction models were determined using Cox regression with age, sex, surgical intervention, diabetes mellitus, tricuspid annular plane systolic excursion (TAPSE), PAPm, carbon monoxide transfer coefficient (KCO [% predicted (pred.)]; DLCO related to alveolar volume), and VO_2_peak (% pred.) as explanatory variables. For the final model, we eliminated variables by a backward selection procedure using a cut-off *p* value of 0.1. The predictive ability of the final model was assessed by Harrell’s C-statistic (concordance index).

Receiver operating characteristic (ROC) curves and the Youden index (defined as sensitivity + specificity −  1) were used to define predictive cut-off values of VO_2_peak (% pred.) for mortality in the surgical and non-surgical groups. Survival rates stratified by these cut-offs were visualized by Kaplan–Meier plots and differences were tested by log-rank tests.

The analyses were performed with Stata 17.0 (Stata Corporation, College Station, TX, USA).

## 3. Results

### 3.1. Patient Characteristics

Of the 345 patients included in the overall study cohort, 138 patients (40.0%) underwent PEA/BPA (surgical group) and 207 patients (60.0%) received only medical therapy (including PAH medication in 168 patients [48.7%]). Patients in the surgical group were significantly younger and had a significantly lower prevalence of arterial hypertension, atrial fibrillation, diabetes mellitus, interstitial lung disease, and peripheral arterial disease than those in the non-surgical group (Table 1).

Table 2 shows functional, echocardiographic, and RHC parameters. The surgical group had better functional capacity in terms of 6-MWD and better left and right ventricular function (left ventricular ejection fraction and TAPSE) than the non-surgical group. RHC data showed no significant differences between the groups.

Lung function and CPET data are shown in Table 3. Compared with the non-surgical group, the surgical group had significantly higher TLC, FVC, and residual volume (RV), but less hyperinflation (RV/TLC). However, diffusion capacity parameters did not differ between the groups. Of the CPET parameters, arterial partial pressure of carbon dioxide (paCO_2_) and PETCO_2_ at rest were significantly lower and VE/VCO_2_ ratio at rest and VE/MVV were significantly higher in the surgical group compared with the non-surgical group.

### 3.2. Survival

**Figure 2:**
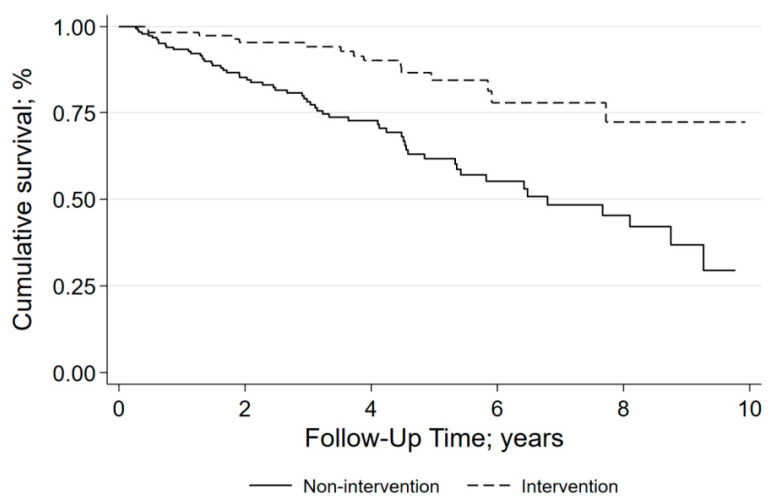
Survival in patients with and without intervention.—Kaplan–Meier curves of cumulative survival stratified by treatment (non-surgical or surgical).

The 345 patients contributed a total of 1532 person-years to the study (follow-up time: median, 3.5 years; mean ± SD, 4.4 ± 3.6 years). Seventy-eight of the 345 patients (22.6%) died (13% of the surgical group and 29% of the non-surgical group), corresponding to 50.9 deaths per 1000 person-years (24.9 per 1000 person-years in the surgical group and 74.2 per 1000 person-years in the non-surgical group; *p* < 0.001). Figure 2 shows the survival curves of the surgical and non-surgical groups.

### 3.3. Determinants of Prognosis

First, we found a significantly lower mortality risk in the surgical group compared with the non-surgical group (HR: 0.51 [95% CI: 0.29–0.89]; *p* = 0.019).

In the second step, we analyzed our data with respect to mortality using Cox regression models adjusted for age and sex in the entire study population as well as in both subgroups (surgical and non-surgical; Table S1).

In the whole study population, we found significant associations of weight, BMI, WHO functional class, diabetes mellitus, TAPSE (as a surrogate of right ventricular function), RVSP estimated by echocardiography, invasively measured PAPm and total pulmonary resistance (TPR), FEV1 (% pred.), DLCO (% pred.), and KCO (% pred.) with mortality. Regarding CPET parameters, we observed significant associations of maximum work rate (% pred.), VO_2_peak, VO_2_/heart rate, VE/VCO_2_ slope, and PETCO_2_ at rest with mortality.

In the non-surgical group, many of the markers that showed significant associations with mortality were the same as in the overall study population, except for diabetes mellitus, TPR, FEV1 (% pred.), and DLCO. Coronary artery disease, pulmonary vascular resistance (PVR), mixed venous oxygen saturation, VE/VCO_2_ ratio at rest and at VT1, PETCO_2_ at rest and at VT1, and P(a-ET)CO_2_ peak were significantly associated with mortality only in the non-surgical group, and not in the whole study cohort.

In the surgical group, only five markers, including the comorbidities chronic obstructive pulmonary disease/asthma, diabetes mellitus, and coronary artery disease, as well as the pulmonary function test markers FEV1/FVC (%) and KCO (% pred.), showed significant associations with mortality.

In the third step, mortality prediction models were calculated by Cox regression. After applying a backward selection procedure in the whole sample, the following markers were kept in the model: age, sex, surgical intervention, BMI, diabetes mellitus, VO_2_ peak (% pred.), and KCO (% pred.). The predictive ability of this model (concordance index) was 0.8066. Using the same procedure in the non-surgical group, age, sex, TAPSE, VO_2_ peak (% pred.), and KCO (% pred.) remained in the model, which had a predictive ability (concordance index) of 0.7722. In the surgical group, only age and BMI were kept in the model, which had a predictive ability (concordance index) of 0.7833.

**Figure 3:**
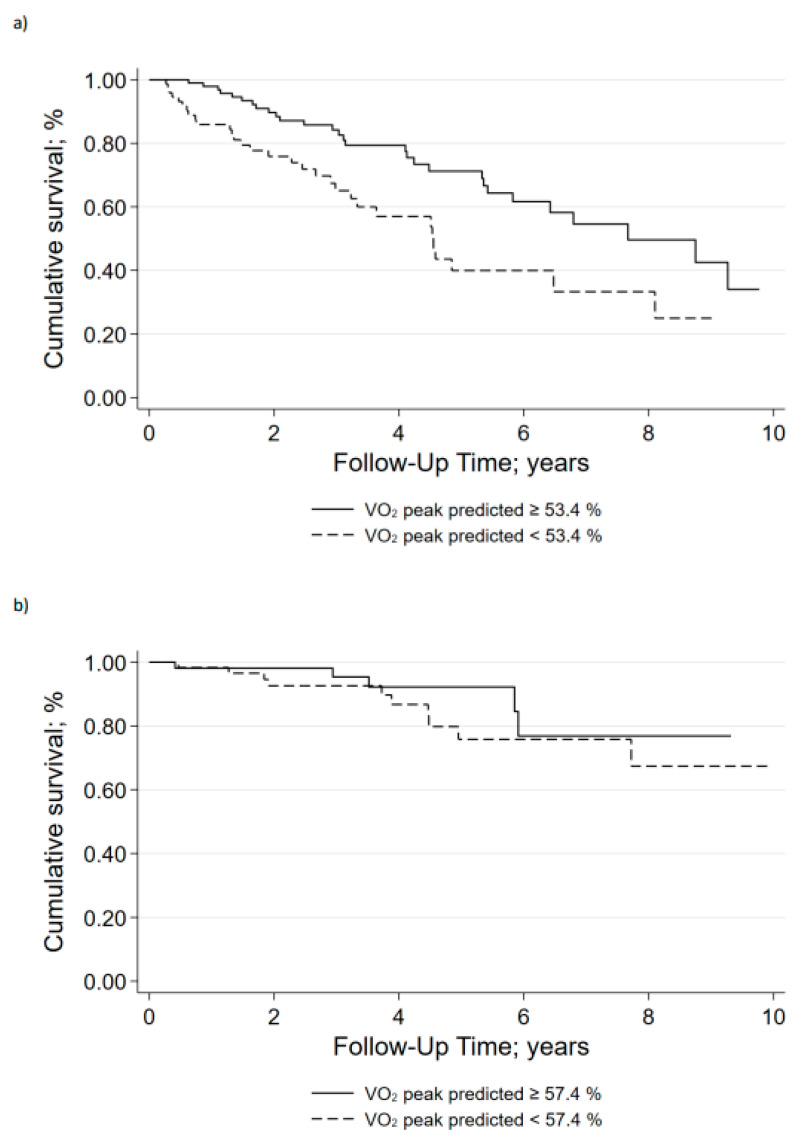
Kaplan-Meier curves for VO_2_peak (% pred.) in (**a**) the non-surgical group and (**b**) the surgical group.—Kaplan–Meier curves of cumulative survival stratified by treatment and VO_2_peak (% predicted). A, Non-surgical group. B, Surgical group. VO_2_peak = peak oxygen uptake.

Predictive cut-off values of VO_2_peak (% pred.) for mortality were determined from ROC curves and the Youden index to be 53.4% pred. in the non-surgical group and 57.4% pred. in the surgical group. Patients with VO_2_peak (% pred.) below the cut-off value had significantly increased mortality in the non-surgical group (*p* = 0.007) but not in the surgical group (*p* = 0.085) (Figure 3).

## 4. Discussion

To our knowledge, this is the first multicenter study showing a significant association between VO_2_peak at the time of diagnosis and long-term survival in CTEPH. Although maximum exercise capacity, measured as VO_2_peak and power, as well as WHO functional class distribution at the time of diagnosis, were comparable in the surgical and non-surgical groups, VO_2_peak was of prognostic importance only in the non-surgical group. Our data suggest that in the surgical group, the treatment (PEA or BPA) has such a strong impact on the underlying pathomechanism (i.e., pulmonary vascular obstruction) that an exercise test at the time of diagnosis cannot predict outcome. By contrast, in the non-surgical group several CPET parameters at diagnosis predicted survival in age- and sex-adjusted Cox regression analysis, including VO_2_peak as well as PETCO_2_ at rest and at VT1, VE/VCO_2_ slope, VE/VCO_2_ at rest and at VT1 (reflecting ineffective ventilation), and P(a-ET)CO_2_ peak (reflecting gas exchange disturbance).

A typical pattern of CPET findings in pulmonary vascular disease is a combination of elevated VE/VCO_2_ slope, elevated breathing equivalents for oxygen and carbon dioxide, and decreased PETCO_2_ indicating hyperventilation and inefficient ventilation [7,9,13]. In our patients, VE/VCO_2_, PETCO_2_, and PaCO_2_, all measured at rest, were more abnormal in the surgical group than in the non-surgical group at diagnosis. Elevated P(A-a)O_2_ and P(a-ET)CO_2_ reflect gas exchange disturbances typically found in CTEPH [7,9,13]. The patients in our surgical and non-surgical groups had comparable values of P(A-a)O_2_, P(a-ET)CO_2_ peak, and PaCO_2_ at maximum exercise. The pathophysiological mechanisms of the abovementioned patterns of hyperventilation, ineffective ventilation, and gas exchange disturbance have recently been discussed in detail and are based, among other factors, on a mismatch of pulmonary ventilation and perfusion and thus increased dead space ventilation [31]. Functional intrapulmonary shunts are significantly involved in the disturbances of gas exchange.

However, although the abovementioned pattern of ineffective ventilation and gas exchange disturbance might help to predict CTEPH [32], detect CTEPH [9], and even distinguish CTEPH from PAH [13], these parameters were not associated with prognosis in our mortality prediction models. None of the pathophysiology-based typical CPET parameters for CTEPH (such as increased VE/VCO_2_ slope, VE/VCO_2_ at VT1, and P[a-ET]CO_2_ or decreased PETCO_2_ at VT1) showed prognostic significance in our regression analysis. This is noteworthy, since impaired ventilatory efficiency in pulmonary vascular diseases has been considered to have a high prognostic significance [33]. Instead, we identified VO_2_peak as a prognostic indicator. Previously, only one single-center study including 53 patients after PE (mostly with PH) had investigated the prognostic role of VO_2_peak (measured on a treadmill as metabolic equivalents), identifying it as an independent risk factor for mortality over a mean follow-up period of 18.7 months [34]. Our results are also consistent with findings in PAH [35,36] and chronic left-sided heart failure [37].

The age and sex distribution of our cohort is consistent with previously published data from other centers or registries [38,39,40]. The occurrence of comorbidities is comparable [41,42] or more frequent [43] in our patients compared with previous studies. This is important because the type and severity of comorbidities can influence outcomes of surgical interventions in CTEPH [8]. Our data also show a lower age and a lower prevalence of certain comorbidities (mainly diabetes, atrial fibrillation, and hypertension) in the surgical group than in the patients treated by medical therapy. This may explain the striking difference in 6-MWD between the surgical and non-surgical groups.

Our finding of better survival in the surgical group than the non-surgical group is consistent with previously published research. Several studies showed significantly better survival following PEA than under medical CTEPH treatment [43]. Patients with inoperable CTEPH treated by BPA also showed an excellent survival of above 90% [44]. Data from the Polish Registry of PH including 516 patients with CTEPH showed superior survival in patients treated by BPA or PEA compared with medically treated individuals [41], whereas there was no difference in survival between the PEA and BPA subgroups.

For patients treated with drug therapy alone, an analysis of COMPERA data was recently performed. In total, 561 patients with CTEPH were included, of whom 231 had a follow-up visit after a median of 7 months [42]. The 1-, 3-, and 5-year survival rate was 92%, 75%, and 60%, respectively. Interestingly, the ERS/ESC risk score, which was actually developed for prognostic assessment of patients with PAH, discriminated prognosis of patients with CTEPH well. However, CPET data were not part of this evaluation.

Previous work has already indicated that submaximal exercise capacity, measured as 6-MWD, is associated with survival following PEA [45,46]. We had rather few data on 6-MWD, which may explain why 6-MWD was not found to be predictive in our study. Hemodynamic parameters such as PVR have also been established as predictors of outcome. PVR and age have repeatedly been shown to have a significant independent effect on mortality after PEA [38]. PVR at rest as well as exercise hemodynamics before PEA correlate with postoperative pulmonary hemodynamics at rest and during exercise [47]. Our mortality prediction models included age but not PVR (the latter was a significant predictor only in the non-surgical group with adjustment for age and sex). In addition to established prognostic parameters such as signs of right heart failure, WHO functional class IV, significant concomitant lung or left-sided heart diseases, high pulmonary artery diastolic pressure, and PVR > 15 Wood Units [8], our results suggest that inclusion of selected CPET parameters could improve the prognostic assessment of patients with CTEPH.

Since we only analyzed patients in whom CPET had been performed at the start of the observation period, our study has a selection bias. Additionally, our study is limited by its retrospective design. However, owing to the multicenter approach a further selection bias can be excluded. Furthermore, all patients underwent a rigorous diagnostic work-up including complete hemodynamic characterization by RHC, and all patients were discussed by a multidisciplinary team including a high-volume CTEPH surgeon before a treatment decision was made. The exercise protocols we chose for CPET are commonly used and validated, as recently reviewed [48]. Two different exercise protocols were used (ramp or Jones protocol); in patients with chronic obstructive pulmonary disease, CPET gas exchange and ventilatory parameters were comparable between these protocols [49,50].

## 5. Interpretation

Based on a multicenter collection of data, we were able to show for the first time that VO_2_peak as a marker of maximum exercise capacity has prognostic relevance in patients with CTEPH receiving non-surgical treatment.

Future analyses should confirm whether VO_2_peak provides additional information compared with previously established prognostic factors. Further studies should also analyze different post-treatment timespans in detail.

## Figures and Tables

**Table 1:** Anthropometric Data and Comorbidities.

Parameter	Non-Surgical Treatment (n = 207)		Surgical Treatment (n = 138)		*p*
	n	Mean ± SD or n (%)	n	Mean ± SD or n (%)	
Age (years)	207	69 ± 12	138	61 ± 12	<0.001
Sex (female)	207	101 (48.8)	138	61 (44.2)	0.403
Body mass index (kg/m^2^)	204	28.7 ± 5.5	137	28.3 ± 4.9	0.675
**Comorbidities**					
Hypertension	194	131 (67.5)	124	66 (53.2)	0.010
Venous thromboembolism	122	75 (61.5)	86	59 (68.6)	0.290
Atrial fibrillation	169	54 (32.0)	112	11 (9.8)	<0.001
Chronic renal failure	193	60 (31.1)	124	37 (29.8)	0.814
Diabetes mellitus	192	37 (19.3)	124	12 (9.7)	0.021
Coronary artery disease	193	37 (19.2)	124	24 (19.4)	0.968
COPD/asthma	139	26 (18.7)	79	12 (15.2)	0.511
Malignancy	160	29 (18.1)	112	16 (14.3)	0.402
Peripheral artery disease	92	6 (6.5)	78	0 (0.0)	0.022
Interstitial lung disease	185	11 (6.0)	122	1 (0.8)	0.023

COPD = chronic obstructive pulmonary disease; SD = standard deviation.

**Table 2:** Functional Capacity, Echocardiography, and Hemodynamic Characteristics.

Parameter	Non-Surgical Treatment (n = 207)		Surgical Treatment (n = 138)		*p*
	n	Mean ± SD or n (%)	n	Mean ± SD or n (%)	
WHO functional class	190		121		
I		5 (2.6)		9 (7.4)	0.071
II		48 (25.3)		39 (32.2)	
III		126 (66.3)		69 (57.0)	
IV		11 (5.8)		4 (3.3)	
Six-minute walk distance (m)	107	336 ± 115	75	390 ± 122	0.001
**Echocardiography**					
LVEF (%)	147	59.9 ± 7.4	95	61.2 ± 10.2	0.031
TAPSE (mm)	151	19.9 ± 5.6	102	19.0 ± 5.7	0.040
Estimated RVSP (mm Hg)	138	59.1 ± 27.1	94	62.4 ± 28.1	0.239
**Right heart catheterization**					
RAPm (mm Hg)	189	8.3 ± 5.0	126	7.9 ± 5.1	0.407
PAPm (mm Hg)	207	41.0 ± 11.1	138	42.2 ± 10.8	0.481
PVR (Wood Units)	179	7.7 ± 4.3	113	7.9 ± 4.2	0.280
TPR (Wood Units)	183	9.9 ± 4.7	111	9.4 ± 3.8	0.321
Cardiac index (L/min/m^2^)	183	2.5 ± 0.8	111	2.4 ± 0.5	0.547
SvO_2_ (%)	140	64.0 ± 8.6	103	63.2 ± 9.3	0.316

LVEF = left ventricular ejection fraction; PAPm = mean pulmonary artery pressure; PVR = pulmonary vascular resistance; RAPm = mean right atrial pressure; RVSP = right ventricular systolic pressure; SD = standard deviation; SvO_2_ = mixed venous oxygen saturation; TAPSE = tricuspid annular plane systolic excursion; TPR = total pulmonary resistance; WHO = World Health Organization.

**Table 3:** Pulmonary Function and Spiroergometric Data.

Parameter	Non-Surgical Treatment (n = 207)		Surgical Treatment (n = 138)		*p*
	n	Mean ± SD	n	Mean ± SD	
**Pulmonary function**					
TLC (% pred.)	178	95.6 ± 17.3	125	101.4 ± 14.4	0.003
FVC (% pred.)	183	86.0 ± 19.8	129	93.8 ± 17.8	0.032
FEV1 (% pred.)	185	80.4 ± 20.3	131	86.5 ± 18.3	0.123
FEV1/FVC (%)	183	72.7 ± 10.4	129	73.8 ± 7.8	0.647
RV (% pred.)	176	114 ± 35	126	120.3 ± 29.4	0.004
RV/TLC (% pred.)	166	47.7 ± 11.7	123	43.0 ± 10.7	0.001
DLCO (% pred.)	105	59.6 ± 18.9	71	64.6 ± 14.7	0.091
KCO (% pred.)	155	74.5 ± 19.9	116	76.1 ± 15.5	0.766
**Cardiopulmonary exercise test**					
Max. power (% pred.)	200	62.2 ± 36.5	129	72.6 ± 42.3	0.159
VO_2_peak (mL/min/kg)	192	12.9 ± 4.2	133	13.2 ± 3.8	0.311
VO_2_peak (% pred.)	192	59.8 ± 19.8	133	57.9 ± 19.7	0.498
VO_2_/heart rate max. (mL/beat)	191	8.8 ± 3.0	132	8.7 ± 2.7	0.857
VE/VCO_2_ slope	164	50.4 ± 16.2	111	53.9 ± 18.1	0.336
VE/VCO_2_ at rest	175	44.6 ± 9.2	111	47.0 ± 9.3	0.026
VE/VCO_2_ at VT1	150	45.0 ± 10.9	100	48.3 ± 12.0	0.352
PETCO_2_ at rest (mm Hg)	173	26.1 ± 5.5	109	24.1 ± 4.5	0.010
PETCO_2_ at VT1 (mm Hg)	147	26.4 ± 6.7	102	24.1 ± 5.9	0.065
P(A-a)O_2_ max. (mm Hg)	100	47.8 ± 14.7	75	51.7 ± 14.5	0.150
P(a-ET)CO_2_ peak (mm Hg)	101	8.3 ± 4.3	74	9.6 ± 4.2	0.057
VE/MVV (%)	163	83.7 ± 29.8	123	103.9 ± 34.7	<0.001
PaO_2_ at rest (mm Hg)	166	65.6 ± 12.8	102	75.6 ± 78.8	0.196
PaO_2_ max. (mm Hg)	144	61.9 ± 13.9	101	62.1 ± 11.0	0.855
PaCO_2_ at rest (mm Hg)	166	35.0 ± 5.2	100	32.9 ± 3.8	0.027
PaCO_2_ max. (mm Hg)	100	34.0 ± 6.9	72	31.6 ± 5.1	0.625

DLCO = diffusion capacity of the lung for carbon monoxide; FEV1 = forced expiratory volume in 1 s; FVC = forced vital capacity; KCO = carbon monoxide transfer coefficient (diffusion capacity of the lung for carbon monoxide related to alveolar volume); max. = maximum; MVV = maximum voluntary ventilation; P(A-a)O_2_ = alveolar to arterial oxygen gradient; PaCO_2_ = arterial partial pressure of carbon dioxide; P(a-ET)CO_2_ = arterial to end-tidal carbon dioxide gradient; PaO_2_ = arterial partial pressure of oxygen; PETCO_2_ = end-tidal partial pressure of carbon dioxide; pred. = predicted; RV = residual volume; TLC = total lung capacity; VCO_2_ = carbon dioxide output; VE = minute ventilation; VO_2_ = oxygen uptake; VO_2_peak = peak oxygen uptake; VT1 = ventilatory threshold 1 (anaerobic threshold).

## Data Availability

Data can be made available with the support of Till Ittermann.

## Supplementary Materials

The following supporting information can be downloaded at: https://www.mdpi.com/article/10.3390/jcdd9100333/s1, Table S1: Cox Regression Analysis Adjusted for Age and Sex.

## Abbreviations

6-MWD = 6-min walk distance; BMI = body mass index; BPA = balloon pulmonary angioplasty; CI = confidence interval; COPD = chronic obstructive pulmonary disease; CPET = cardiopulmonary exercise testing; CTEPD = chronic thromboembolic pulmonary disease; CTEPH = chronic thromboembolic pulmonary hypertension; DLCO = diffusion capacity of the lung for carbon monoxide; ERS = European Respiratory Society; ESC = European Society of Cardiology; FEV1 = forced expiratory volume in 1 s; FVC = forced vital capacity; HR = hazard ratio; KCO = carbon monoxide transfer coefficient (diffusion capacity of the lung for carbon monoxide related to alveolar volume); LVEF = left ventricular ejection fraction; max. = maximum; MVV = maximum voluntary ventilation; P(A-a)O_2_ = alveolar to arterial oxygen gradient; PaCO_2_ = arterial partial pressure of carbon dioxide; P(a-ET)CO_2_ = arterial to end-tidal carbon dioxide gradient; PAH = pulmonary arterial hypertension; PaO_2_ = arterial partial pressure of oxygen; PAPm = mean pulmonary artery pressure; PE = pulmonary embolism; PEA = pulmonary endarterectomy; PETCO_2_ = end-tidal partial pressure of carbon dioxide; PH = pulmonary hypertension; pred. = predicted; PVR = pulmonary vascular resistance; RAPm = mean right atrial pressure; RHC = right heart catheterization; ROC = receiver operating characteristic; RV = residual volume; RVSP = right ventricular systolic pressure; SD = standard deviation; SvO_2_ = mixed venous oxygen saturation; TAPSE = tricuspid annular plane systolic excursion; TLC = total lung capacity; TPR = total pulmonary resistance; VCO_2_ = carbon dioxide output; VE = minute ventilation; VO_2_ = oxygen uptake, VO_2_peak = peak oxygen uptake; VT1 = ventilatory threshold 1 (anaerobic threshold); WHO = World Health Organization.
