# Supplementary material for: Prognostic Relevance of Cardiopulmonary Exercise Testing for Patients with Chronic Thromboembolic Pulmonary Hypertension
Source: J Cardiovasc Dev Dis. 2022 Oct 1;9(10):333. doi: 10.3390/jcdd9100333 (PMC9604581; doi:10.3390/jcdd9100333)
Supplement: Supplementary file 1 [file jcdd-09-00333-s001.zip › jcdd-1909887-supplementary.pdf]

Supplemental Material

Prognostic Relevance of Cardiopulmonary Exercise Testing for Patients With Chronic Thromboembolic Pulmonary Hypertension

Table S1. Cox Regression Analysis Adjusted for Age and Sex.

Parameter	n	Total Population HR (95% CI)	Non-Surgical Treatment (n = 207) HR (95% CI)	Surgical Treatment (n = 138) HR (95% CI)
**Height**	341	0.92 (0.68; 1.26)	0.91 (0.63; 1.29)	1.38 (0.72; 2.66)
**Weight**	345	0.63 (0.47; 0.84)*	0.63 (0.46;0.87)*	0.67 (0.36; 1.28)
**Body mass index**	341	0.63 (0.47; 0.84)*	0.62 (0.45; 0.86)*	0.57 (0.30; 1.07)
**PAH medical treatment**	345	0.92 (0.58; 1.47)	0.88 (0.51; 1.49)	0.56 (0.19; 1.64)
**WHO functional class at diagnosis**	311			
III & IV vs I & II		2.67 (1.40; 5.08)*	2.58 (1.22; 5.48)*	2.56 (0.73; 8.95)
**6-MWD at diagnosis**	182	0.74 (0.54; 1.03)	0.77 (0.53; 1.14)	0.73 (0.34; 1.56)
**Comorbidities**				
Arterial hypertension	318	0.68 (0.43; 1.10)	0.63 (0.37; 1.09)	0.87 (0.32; 2.34)
Venous thromboembolism	208	1.27 (0.68; 2.35)	0.91 (0.46; 1.81)	3.15 (0.65; 15.31)
Atrial fibrillation	281	0.82 (0.49; 1.38)	0.86 (0.50; 1.48)	0.36 (0.04; 2.94)
Chronic renal failure	317	1.30 (0.81; 2.08)	1.12 (0.64; 1.94)	2.05 (0.78; 5.41)
Diabetes mellitus	316	1.84 (1.05; 3.21)*	1.43 (0.75; 2.72)	3.49 (1.06; 11.53)*
Coronary artery disease	317	1.08 (0.65; 1.82)	1.90 (1.08; 3.34)*	0.20 (0.04; 0.93)*
COPD/asthma	218	1.29 (0.65; 2.54)	0.88 (0.38; 2.01)	4.12 (1.12; 15.18)*
Malignancy	272	0.81 (0.44; 1.49)	0.76 (0.38; 1.53)	1.07 (0.28; 3.99)
Peripheral artery disease	170	2.12 (0.80; 5.60)	1.87 (0.71; 4.95)	-
Interstitial lung disease	307	2.15 (0.86; 5.40)	2.05 (0.80; 5.26)	-
**Echocardiography**				
LVEF	242	0.90 (0.66; 1.22)	0.90 (0.61; 1.34)	0.98 (0.56; 1.72)
TAPSE	253	0.70 (0.53; 0.91)*	0.69 (0.51; 0.94)*	0.76 (0.34; 1.35)
Estimated RVSP	232	1.30 (1.01; 1.66)*	1.47 (1.08; 2.00)*	1.05 (0.65; 1.70)
**Right heart catheterization**				
RAPm	315	1.15 (0.91; 1.46)	1.22 (0.93; 1.61)	0.92 (0.54;1.58)

Parameter	n	Total Population<br>HR (95% CI)	Non-Surgical Treatment (n = 207)<br>HR (95% CI)	Surgical Treatment (n = 138)<br>HR (95% CI)
PAPm	345	1.33 (1.08; 1.64)*	1.41 (1.12; 1.78)*	1.03 (0.63; 1.70)
PVR	292	1.21 (0.97; 1.51)	1.29 (1.01; 1.67)*	0.98 (0.60; 1.61)
TPR	294	1.30 (1.04; 1.61)*	1.26 (0.99; 1.60)	1.25 (0.75; 2.08)
Cardiac index	294	0.77 (0.58; 1.02)	0.79 (0.59; 1.07)	0.73 (0.37; 1.43)
SvO_2	243	0.87 (0.69; 1.11)	0.61 (0.43; 0.86)*	1.20 (0.66; 2.19)
**Pulmonary function test**				
TLC (% pred.)	303	0.89 (0.70; 1.13)	0.96 (0.74; 1.25)	0.99 (0.53; 1.86)
FVC (% pred.)	312	0.81 (0.64; 1.03)	0.87 (0.67; 1.15)	0.81 (0.47; 1.40)
FEV1 (% pred.)	316	0.73 (0.57; 0.93)*	0.81 (0.62; 1.06)	0.62 (0.35; 1.08)
FEV1/FVC (%)	312	0.79 (0.61; 1.01)	0.89 (0.68; 1.18)	0.51 (0.30; 0.89)*
RV (% pred.)	302	1.08 (0.87; 1.35)	1.10 (0.87; 1.39)	1.31 (0.79; 2.17)
RV/TLC (% pred.)	289	1.18 (0.92; 1.52)	1.05 (0.79; 1.41)	2.00 (0.93; 4.29)
DLCO (% pred.)	176	0.69 (0.51; 0.93)*	0.74 (0.53; 1.01)	0.75 (0.37; 1.53)
KCO (% pred.)	271	0.60 (0.48; 0.75)*	0.64 (0.51; 0.82)*	0.54 (0.31; 0.92)*
**Cardiopulmonary exercise testing**				
Max. work rate (% pred.)	325	0.62 (0.47; 0.84)*	0.63 (0.44; 0.91)*	0.64 (0.36; 1.14)
VO_2 peak/kg	328	0.65 (0.49; 0.86)*	0.59 (0.42; 0.84)*	0.74 (0.43; 1.28)
VO_2 peak (% pred.)	328	0.55 (0.41; 0.75)*	0.49 (0.34; 0.70)*	0.61 (0.33; 1.12)
VO_2/heart rate max.	323	0.62 (0.47; 0.82)*	0.63 (0.46; 0.87)*	0.54 (0.29; 1.01)
VE/VCO_2 slope	275	1.32 (1.03; 1.68)*	1.59 (1.16; 2.18)*	1.11 (0.69; 1.79)
VE/VCO_2 at rest	286	1.26 (0.99; 1.59)	1.40 (1.04; 1.87)*	1.12 (0.73; 1.71)
VE/VCO_2 at VT1	250	1.18 (0.94; 1.49)	1.41 (1.03; 1.95)*	1.02 (0.67; 1.55)
$PETCO_2$ at rest	282	0.78 (0.61; 0.99)*	0.75 (0.56; 0.99)*	0.77 (0.46; 1.31)
$PETCO_2$ at VT1	249	0.77 (0.59; 1.01)	0.72 (0.52; 0.99)*	0.78 (0.46; 1.33)
$P(A-a)O_2$ max.	175	1.14 (0.86; 1.52)	1.06 (0.74; 1.50)	1.47 (0.84; 2.58)

Parameter	n	Total Population<br>HR (95% CI)	Non-Surgical Treatment (n = 207)<br>HR (95% CI)	Surgical Treatment (n = 138)<br>HR (95% CI)
$P(a\text{-}ET)CO_2$ peak	175	1.27 (0.96; 1.69)	1.39 (1.01; 1.91)*	1.18 (0.61; 2.29)
VE/MVV (%)	286	0.71 (0.49; 1.03)	0.85 (0.55; 1.30)	0.70 (0.32; 1.53)
PaO_2 at rest	268	0.54 (0.18; 1.61)	0.60 (0.18; 1.99)	0.59 (0.05; 6.82)
PaO_2 max.	245	0.84 (0.65; 1.09)	0.89 (0.66; 1.19)	0.66 (0.38; 1.17)
$PaCO_2$ at rest	266	1.07 (0.83; 1.37)	1.13 (0.86; 1.47)	0.77 (0.41; 1.45)
$PaCO_2$ max.	172	0.86 (0.63; 1.17)	0.86 (0.60; 1.24)	0.82 (0.43; 1.57)

For continuous exposures, HRs are expressed for a change of 1 standard deviation.

6-MWD = six-minute walk distance; CI = confidence interval; DLCO = diffusion capacity of the lung for carbon monoxide; FEV1 = forced expiratory volume in 1 second; FVC = forced vital capacity; HR = hazard ratio; KCO = carbon monoxide transfer coefficient (diffusion capacity of the lung for carbon monoxide related to alveolar volume); LVEF = left ventricular ejection fraction; max. = maximum; MVV = maximum voluntary ventilation; $P(A\text{-}a)O_2$ = alveolar to arterial oxygen gradient; $PaCO_2$ = partial pressure of carbon dioxide; $P(a\text{-}ET)O_2$, arterial to end-tidal carbon dioxide gradient; PAH = pulmonary arterial hypertension; PaO_2 = partial pressure of oxygen; PAPm = mean pulmonary artery pressure; $PETCO_2$ = end-tidal partial pressure of carbon dioxide; pred. = predicted; PVR = pulmonary vascular resistance; RAPm = mean right atrial pressure; RV = residual volume; RVSP = right ventricular systolic pressure; SvO_2 = mixed venous oxygen saturation; TAPSE = tricuspid annular plane systolic excursion; TLC = total lung capacity; TPR = total pulmonary resistance; VCO_2 = carbon dioxide output; VE = minute ventilation; VO_2 = oxygen uptake; VT1 = ventilatory threshold 1 (anaerobic threshold); WHO = World Health Organization.

*$P < .05$
